# Quality of life in type 2 and non‐type 2 endotypes in chronic rhinosinusitis with nasal polyps: A prospective trial

**DOI:** 10.1002/clt2.70070

**Published:** 2025-05-31

**Authors:** Stijn Bogaert, Elisabeth Rheindorf, Stefan Dazert, Stefan Volkenstein, Lisa Knipps, Jonas Jae‐Hyun Park, Oliver Pfaar

**Affiliations:** ^1^ Department of Otorhinolaryngology Head and Neck Surgery St. Elisabeth‐Hospital Ruhr University Bochum Bochum Germany; ^2^ Department of Otorhinolaryngology Head and Neck Surgery Johannes Wesling Medical Center Minden Ruhr University Bochum Minden Germany; ^3^ Department of Otorhinolaryngology Head and Neck Surgery St. Josef‐Hospital Witten/Herdecke University Hagen Germany; ^4^ Department of Otorhinolaryngology Head and Neck Surgery Section of Rhinology and Allergy Philipps‐Universität Marburg University Hospital Marburg Marburg Germany

**Keywords:** biomarkers, chronic rhinosinusitis, nasal polyps, quality of life, sino‐nasal outcome test

## Abstract

**Background:**

In current clinical practice, primary diffuse chronic rhinosinusitis with nasal polyps (CRSwNP) is classified into two endotypes: type 2 and non‐type 2. Previous studies on sinonasal health‐related quality of life (HRQoL) in CRS have primarily focused on differences between phenotypes. This study aimed to compare HRQoL between the two endotypes in patients with CRSwNP.

The type 2 endotype had a higher median nasal polyp score (NPS) than non‐types (4 and 2, respectively), but this difference did not reach significance. Loss of smell was associated with NPS, and facial pain/pressure was inversely correlated with age. Age was significantly associated with loss of smell, but only in non‐type 2 CRSwNP.

**Methods:**

This was a prospective, monocentric study conducted between 2018 and 2023 on CRSwNP patients referred for surgery. Health‐related quality of life was assessed using the German standardized SNOT‐20 questionnaire. Type 2 was defined according to the updated EPOS/EUFOREA 2023 criteria.

**Results:**

A total of 122 patients with CRSwNP were included, 113 (92.6%) of whom were classified as type 2. Type 2 was associated with a significantly worse SNOT‐20 German Adapted Version score. Two of the four cardinal symptoms of CRS—loss of smell and rhinorrhea—were significantly more severe and prevalent in the type 2 endotype, with loss of smell being very specific. The most prevalent symptom in both endotypes was nasal obstruction, with no difference between both endotypes.

The type 2 endotype had a higher median nasal polyp score (NPS) than non‐types (4 and 2, respectively), but this difference did not reach significance. Loss of smell was associated with NPS, and facial pain/pressure was inversely correlated with age. Age was significantly associated with loss of smell, but only in non‐type 2 CRSwNP.

**Conclusion:**

Type 2 CRSwNP has a more severe impact on HRQoL compared with non‐type 2 CRSwNP. Hyposmia, rhinorrhea, and potentially NPS may offer endotypic and pathophysiological insights.

## 1 BACKGROUND

Primary diffuse chronic rhinosinusitis (CRS) is a prevalent chronic condition that significantly impairs health‐related quality of life (HRQoL).^1^, ^2^ The cardinal symptoms (CS) defining CRS include nasal obstruction, rhinorrhea (anterior/posterior nasal drip), facial pain/pressure, and a reduction in the sense of smell.^1^ Traditionally, primary diffuse CRS has been categorized into two phenotypes based on rhinoscopic findings: CRS with nasal polyps (CRSwNP) and CRS without nasal polyps (CRSsNP).^3^ However, the development of nasal polyps can result from diverse underlying pathophysiological mechanisms. Tomassen et al. identified 10 CRS clusters based on biomarkers in the sinonasal mucosa.^4^ However, their clinical significance and impact on HRQoL remain unclear. Clusters characterized by heightened T_H_2 inflammation are associated with a higher prevalence of CRSwNP, asthma and responsiveness to anti‐inflammatory treatments, including corticosteroids and biologics.^4^, ^5^ In current clinical practice, primary diffuse CRS is currently classified into two endotypes: type 2 and non‐type 2.^1^ Approximately 85% of CRSwNP patients exhibit type 2 mucosal inflammation, although prevalence varies across regions and ethnic groups.^4^, ^6^ Both phenotype and endotype are clinically relevant, as biological agents are indicated only for severe, uncontrolled CRSwNP with evidence of type 2 inflammation.^7^ Type 2 inflammation is defined by either a serum total IgE >100 kU/L, a blood eosinophil count ≥150 cells/μL, or a tissue eosinophil count ≥10 cells per high‐power field (HPF), as updated by EPOS/EUFOREA in 2023.^7^

Sinonasal quality of life is frequently assessed by a Sino‐Nasal Outcome Test (SNOT) questionnaire, most commonly SNOT‐22.^8^, ^9^ In Germany, SNOT‐20 German Adapted Version (GAV) has been introduced and validated for several years.^10^, ^11^ While the SNOT questionnaires encompass the major CRS symptoms, they also include general HRQoL symptoms, making them non‐specific to CRS. The SNOT‐20 GAV can be divided into three subdomains: primary nasal symptoms (PNS), secondary rhinologic symptoms (SRS) and general symptoms of quality of life (GQL). Previous HRQoL studies in CRS have primarily focused on phenotypic differences, revealing no significant difference in total scores between CRSwNP and CRSsNP.^12^, ^13^, ^14^ However, patients with CRSwNP have been reported to experience more severe and frequent nasal obstruction, alterations in the sense of smell, and rhinorrhea compared with those with CRSsNP. Therefore, this study aimed to compare sinonasal quality of life between the two endotypes in patients with CRSwNP. Additionally, we wanted to investigate an association between endotype and Nasal Polyp Score (NPS) in CRSwNP.

## 2 METHODS

### 2.1 Patients and sample collection

All consecutive patients aged 18 years or older with CRSwNP, who had persistent symptoms despite appropriate medical therapy–including ≥8 weeks of mometasone furoate nasal spray at the maximum dosage of 400 μg per day–and were referred for sinus surgery, were prospectively included at the Department of Otolaryngology, Ruhr‐University Hospital Bochum, Germany. Routine clinical histopathological examination was used to exclude secondary CRSwNP. Patients with unilateral disease, immunodeficiency, or incomplete SNOT‐20 questionnaires were excluded. Oral corticosteroids were discontinued at least 4 weeks prior to surgery and nasal corticosteroids at least 2 weeks preoperatively. Inhalant allergies and aspirin intolerance were obtained from the patient's medical history. The diagnosis of asthma was based on the GINA guidelines.^15^ The inclusion period was between September 2018 and August 2023. Because of the COVID‐19 pandemic, no patients were included between March 2020 and February 2023. The study was conducted in accordance with the principles of the Declaration of Helsinki (2000) and the EMA Guidelines for Good Clinical Practice (2017). The study was approved by the local ethical committee (registration number 17–6245) and written informed consent was obtained from all patients.

The a priori calculation using G*Power 3.1 (Heinrich Heine University Düsseldorf, Düsseldorf, Germany), assuming an allocation ratio based on 90% type 2 CRSwNP in our study population, suggested a minimum sample size of 98 to achieve a statistical power of 95% and a type I error rate <0.05.^12^, ^16^

### 2.2 Definitions and data collection

Diagnosis of CRSwNP was established based on patient history, clinical symptoms, nasal endoscopy and CT imaging, in accordance with the 2020 EPOS guidelines.^1^ CRSwNP was defined as primary CRS with bilateral disease, characterized by endoscopically confirmed bilateral nasal polyps in the middle meatus, identified either preoperatively or intraoperatively. Sinonasal HRQoL was assessed using the SNOT‐20 GAV, a validated questionnaire with a maximum score of 100.^10^ This 20‐item questionnaire is structured into three subdomains: PNS (5 items), SRS (6 items), and GQL (9 items). Additionally, scores for the four CS of CRS were also calculated as percentages.^1^ Severe CRS is defined as an SNOT‐20 GAV score ≥33. In alignment with studies by Dietz de Loos et al. and Abdullah et al., symptom scores were dichotomized into positive outcomes for the respective symptom based on a score ≥2 (i.e., mild or worse).^12^, ^14^ Therefore, a symptom was considered as present when the symptom score was ≥2.

Polyp size was scored endoscopically by one of the study doctors to determine the NPS following the classification of the European Academy of Allergy and Clinical Immunology (EAACI).^17^ This scale ranges from 0 (no polyp) to 4 (polyps obstructing the inferior meatus) for each nostril. The scores from each nostril are summed for a total score ranging from 0 to 8. Paranasal CT scans were scored using the Lund–Mackay scoring system (0–24).^1^, ^18^

Markers for type 2 inflammation were defined as tissue eosinophil count ≥10/HPF, blood eosinophil count ≥150/μL, or total IgE ≥100 kU/l.^7^ The tissue eosinophil count was calculated as the mean number of eosinophils per high power field (HPF, ×400) from five randomly selected fields on nasal polyp samples. The examination was conducted by an experienced histotechnologist who was blinded to patient data. The eosinophil count in whole blood and IgE in serum were routinely analyzed in the central laboratory of our university hospital.

After data collection was merged in Microsoft Excel (Microsoft Corp., Redmond, WA), both automated and manual data verification processes were performed. The raw dataset is available in a public repository.^19^ Statistical analyses were conducted using SPSS software version 25.0 (SPSS Inc., Chicago, Illinois, USA).

### 2.3 Data analysis

All statistical analyses were conducted using the SPSS statistical package. The Kolmogorov–Smirnov and Shapiro–Wilk tests were utilized to assess the normality of variable distribution. To compare continuous variables between the two groups, we used either the Mann–Whitney *U* test or an unpaired *t*‐test. Univariate correlations between continuous variables were examined using Pearson's chi‐square test. Categorical variables were presented as counts (percentages) and were compared using Spearman's rank correlation coefficient or Fisher's exact test. Predictive values of type 2 comorbidities for identifying type 2 inflammation in CRSwNP were calculated using Bayes' theorem based on comparison with current biomarker thresholds and point prevalence estimates of 46.9% for asthma, 31.0% for allergic rhinitis, and 9.6% for aspirin sensitivity in CRSwNP.^20^ The positive likelihood ratio for aspirin sensitivity could not be calculated because of its 100% specificity for the type 2 endotype in our study population. A two‐sided *p*‐value of less than 0.05 was considered statistically significant. Radar charts were generated using Microsoft Excel (Microsoft Corp., Redmond, WA, USA).

## 3 RESULTS

A total of 122 patients with primary diffuse CRS with the CRSwNP phenotype met the inclusion and exclusion criteria. Endotype 2 was found in 113 (92.6%) patients. The demographic and clinical characteristics of the patients are listed in Table 1. The mean SNOT‐20 GAV score was 38.4 and the CRS was considered severe in 57.4% of the patients. Type 2 was associated with a significantly worse SNOT‐20 GAV score (mean of 39.4 vs. 26.7, *p* = 0.01). CS were also significantly higher in type 2 (mean of 56% vs. 20%, *p* < 0.001). Within the SNOT‐20 GAV subdomains, type 2 patients had higher PNS scores (*p* = 0.003) but no significant differences in SRS or GQL (Table 2).

**TABLE 1:** Patient characteristics.

Variables	Type 2	Non‐type 2
*N*	113 (92.6%)	9 (7.4%)
Mean age, y	51.4 (SD 14.2)	51.0 (SD 20.5)
Female gender	37 (33.0%)	3 (33.3%)
Nicotine use	21 (18.6%)	1 (14.3%)
Previous ESS	48 (42.5%)	0
1 previous ESS	41 (36.3%)	0
≥2 previous ESS	7 (6.2%)	0
Asthma	43 (38.4%)	1 (11.1%)
Allergic rhinitis	45 (40.9%)	1 (11.1%)
Aspirin sensitivity	14 (13.0%)	0
BMI, kg/m^2^	26.6 [23.8–29.9]	26.5 [23.7–27.5]
LM	12 [8–17]	12 [6–17]
NPS	4 [2–5]	2 [1–4]
Tissue eosinophil count,/HPF	58.0 (SD 72.9)	1.0 (SD 1.2)
Blood eosinophil count,/μl	392.7 (SD 268.0)	94.4 (SD 33.2)
Total IgE, kU/l	145 [74–330]	29 [4–50]

*Note*: Amounts are depicted as *n* (%), normal variables as mean (SD) and non‐normally distributed continuous data as median [IQR].

Abbreviations: BMI, body mass index; HPF, high‐power field; IQR, interquartile range; LM, Lund–Mackay score; NPS, nasal polyp score; SD, standard deviation.

**TABLE 2:** Symptom scores in type 2 and non‐type 2 CRSwNP.

Symptom scores	Type 2	Non‐type 2	*p*‐value
SNOT‐20 GAV	39.4 (SD 17.6)	26.7 (SD 11.4)	0.01
Severe	66 (58.4%)	4 (44.4%)	
Subdomains			
CS, %	56 [40–68]	20 [20–40]	<0.001
PNS, %	64 [44–76]	36 [24–44]	0.003
SRS, %	32 [20–48]	27 [10–37]	0.09
GQL, %	31 [11–49]	27 [6–38]	0.3
CS and PNS symptoms			
Nasal obstruction	4 [3–5]	3 [2–4.5]	0.2
Rhinorrhea	3 [1–4]	0 [0–0]	<0.001
Postnasal drip	2 [1–4]	2 [2–4]	0.8
Reduced sense of smell	5 [3–5]	1 [0.5–3.5]	0.002
Facial pain/pressure	0 [0–3]	0 [0 ‐ 0]	0.03
Sneezing	2 [1–3]	2 [0–2.5]	0.2
Thick nasal discharge	3 [2–4]	3 [1–4]	0.4

*Note*: Amounts are depicted as *n* (%), normal deviates as mean (SD) and ordinal data as median [IQR].

Abbreviations: CS, cardinal symptoms of CRS; GQL, general quality of life score; PNS, primary nasal symptoms; SRS, secondary rhinological symptoms.

**FIGURE 1:**
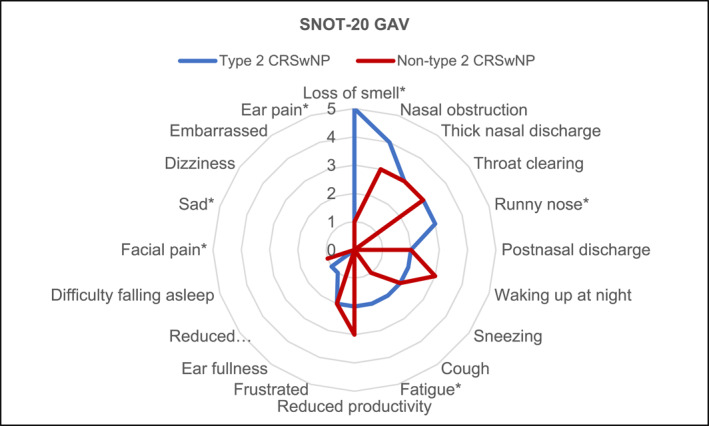
Radar chart of the median sinonasal symptom scores in CRSwNP with the type 2 (A) and non‐type 2 (B) endotypes. Symptom scores are ordered in descending order as for type 2 CRSwNP. Symptoms marked with an asterisk (*) are significantly worse in type 2 CRSwNP. CRSwNP, chronic rhinosinusitis with nasal polyps.

The highest median symptom scores in endotype 2 CRSwNP were for reduced sense of smell (median 5), followed by nasal obstruction (median 4), rhinorrhea, thick nasal discharge and throat clearing (median 3). In non‐type 2 CRSwNP, the most severe nasal symptoms were nasal obstruction and thick nasal discharge (both median 3). There was no endotype‐specific difference in the nasal obstruction score. Severe nasal symptoms that significantly differed between type 2 and non‐type 2 CRSwNP were rhinorrhea (median of 3 vs. 0, *p* < 0.001) and reduced sense of smell (median of 5 vs. 1, *p* = 0.002). The median SNOT‐20 GAV symptom scores in both endotypes are displayed in a radar chart (Figure 1).

Nasal obstruction was the most prevalent symptom, occurring in 94.7% of patients with type 2 CRSwNP and 88.9% of those with non‐type 2 CRSwNP (*p* = 0.4). Patients with type 2 CRSwNP suffered significantly more often from reduced sense of smell (83.2% vs. 44.4%, *p* = 0.01) and rhinorrhea (83.2% vs. 0.0%, *p* < 0.001) (Table S1). Facial pain was neither severe nor prevalent in both endotypes (median score of 0 in both groups).

The type 2 endotype had a higher median NPS than the non‐type (4 and 2, respectively) but this difference did not reach significance (*p* = 0.2). Type 2 inflammation was also not associated with the LM‐score (*p* = 0.6). In the type 2 endotype, NPS was significantly associated with nasal obstruction (*p* = 0.003), rhinorrhea (*p* = 0.001), reduced sense of smell (*p* < 0.001), and sneezing (*p* = 0.04), whereas no significant associations were observed in patients with non‐type 2 CRSwNP. Age was significantly associated with reduced sense of smell in patients with non‐type 2 CRSwNP (*ρ* = 0.8, *p* = 0.01) but not in type 2 CRSwNP (*p* = 0.5). In type 2 CRSwNP, facial pain/pressure showed a significant inverse correlation with age (*p* = 0.01, *ρ* = −0.25, Figure S1). The type 2 group had higher rates of comorbid asthma (38.4%) and allergic rhinitis (40.9%) than the non‐type 2 group (11.1%), but these differences did not reach statistical significance (*p* = 0.1). The positive likelihood ratio of comorbid asthma for type 2 inflammation was 3,5. The predictive values of comorbid type 2 comorbidities for identifying type 2 CRSwNP are listed in Table 3.

**TABLE 3:** Diagnostic performance of type 2 comorbidities for predicting type 2 inflammation in CRSwNP.

Comorbidity	PPV (%)	NPV (%)	Sensitivity (%)	Specificity (%)	LR+
Asthma	48.1	84.3	38.4	88.9	3.46
AR	48.4	85.5	40.9	88.9	3.68
ASSIntoleranz	100.0	91.5	13.0	100	‐^a^

Abbreviations: LR +, positive likelihood ratio; NPV, negative predictive value; PPV, positive predictive value.

^a^ The positive likelihood ratio for aspirin sensitivity could not be calculated because of its 100% specificity for the type 2 endotype in our study population.

Comorbid allergic rhinitis was significantly associated with worse rhinorrhea scores (median score of 3 vs. 2, *p* = 0.01). Among patients with CRSwNP without allergic rhinitis, those with type 2 still reported significantly worse rhinorrhea scores compared with non‐type 2 CRSwNP (median 2 vs. 0, *p* < 0.001). House dust mite allergic rhinitis was not associated with differences in nasal obstruction scores but was significantly linked to more difficulties falling asleep (median score of 2 vs. 0, *p* = 0.04).

## 4 DISCUSSION

This study is the first to examine the impact of the current endotypes on symptom severity in CRSwNP. The endotype of CRSwNP has a significant impact on quality of life and specific nasal symptoms. Patients with type 2 CRSwNP have a significantly worse SNOT‐20 GAV (*p* = 0.01), which supports previous findings from Macchi et al. on SNOT‐22.^21^ This Italian study by Macchi et al., however, did not investigate symptoms separately and defined type 2 as BEC ≥250 cells/μL or serum total IgE >100 kU/L. In our study, type 2 CRSwNP patients had significantly higher CS scores (*p* < 0.001) compared with non‐type 2 CRSwNP.

Loss of smell was the most severe symptom in patients with type 2 CRSwNP, with a significant difference compared to non‐type 2 patients (median of 5 vs. 1). Rhinorrhea was another severe and specific CS for endotype 2 (median of 3 vs. 0), independent of comorbid allergic rhinitis. Nasal obstruction was the most severe symptom in non‐type 2 CRSwNP, with no significant difference from type 2. Facial pain, although a CS, was not a prominent symptom in either endotype. The most prevalent symptom in both type 2 and non‐type 2 CRSwNP was nasal obstruction (95% and 89%, respectively) with no significant difference between both endotypes. However, reduced sense of smell and rhinorrhea were both frequent symptoms in type 2 CRSwNP and were significantly more prevalent than in non‐type 2 CRSwNP.

Hyposmia is the most severe symptom of type 2 CRSwNP and nasal obstruction is the most prevalent symptom. This finding aligns with previous studies by Dietz de Loos et al. and Abdalla et al., which investigated the phenotype CRSwNP with a predominance of type 2 patients.^12^, ^14^ Patients included in phase 3 trials on dupilumab for CRSwNP ranked decreased sense of smell/taste as most important at baseline, followed by nasal blockage.^22^, ^23^

In our study, the type 2 endotype had a higher median NPS than the non‐type 2 endotype (4 vs. 2, respectively), though this difference did not reach statistical significance. In contrast, Macchi et al. included 811 patients and, although using different threshold biomarker values for type 2, found a significant difference (4.6 vs. 3.0, *p* < 0.0001).^21^ Our study found no association between NPS and hyposmia in non‐type 2 CRSwNP. This supports the idea that hyposmia in CRSwNP is mainly driven by local inflammation rather than polyps obstructing the nasal airflow. Conversely, in type 2 CRSwNP, the sense of smell worsened with increasing polyp size. These findings suggest that the polyp size itself does not have a large influence on smell but might be an indirect marker for the severity of the local type 2 inflammation, which causes the loss of smell. Olfaction is known to be influenced by aging as well.^24^ Indeed, this association was strong in patients with non‐type 2 CRSwNP. In type 2 CRSwNP, by contrast, smell was mainly reduced by the local inflammation and age did not have an additional influence. There was no difference in age between both endotypes. Our study confirms the association between house dust mite allergic rhinitis and impaired sleep quality in adults with CRSwNP.^25^

Chronic rhinosinusitis is known to affect extranasal symptoms and mental health as well.^26^, ^27^ Anosmia itself might also have a direct association with scores for anxiety, phobia and depression in CRSwNP.^28^ The SNOT questionnaires are a validated tool to assess sinonasal HRQoL in CRS. Although these questionnaires comprehensively capture CS, they also encompass other (extra)nasal symptoms and general HRQoL‐related aspects, which may be considered a disadvantage due to their limited specificity for CRS. Every symptom in the questionnaire is equally scored and the majority of the questions are about secondary nasal and even general symptoms, such as coughing and sleeping problems, which are more often caused by causes other than CRS. Another advantage of the SNOT questionnaires is their validation in multiple languages and therefore international comparability. More recently, the SNOT‐22 questionnaire has been validated in German as well.^11^, ^29^

Nasal obstruction, rhinorrhea and reduction of smell are all CS in the definition of CRS. The other CS items, postnasal drip and facial pain, were neither severe nor prevalent symptoms in either endotype of CRSwNP. The subdomain PNS is not CRS‐specific and contains symptoms different from the CS of CRS. Both PNS and CS include nasal obstruction and reduced sense of smell. However, PNS also includes sneezing and thick nasal discharge, which are not major symptoms for CRS. Interestingly, these symptoms were more severe and more prevalent than postnasal drip and facial pain/pressure in both endotypes of CRSwNP.

A shorter and more targeted alternative is to semi‐quantify only the clinically relevant sinonasal symptoms of active disease. Experts from EPOS2020/EUFOREA proposed using a visual analog scale (VAS) for overall symptom severity, nasal obstruction, and loss of smell to evaluate disease control.^30^ Our study confirmed that the CS are by far the most severe and prevalent symptoms of CRSwNP. This supports the use of limited, more specific questionnaires to quantify the symptom severity in CRS in light of specific diease‐burden. Our study, however, also showed that the prevalence of these symptoms is endotype‐dependent. Nasal obstruction is a frequent and severe symptom of both CRSwNP endotypes. Loss of smell but also rhinorrhea, by contrast, are very specific for the type 2 endotype. This finding suggests that adding a VAS score for rhinorrhea in assessing disease control in CRSwNP might provide additional endotype‐specific and therefore pathophysiological information.

The classification of primary diffuse CRS into type 2 and non‐type 2 inflammation in this study adheres to the current EPOS/EUFOREA guidelines, which are widely considered the gold standard for the diagnosis and management of CRS in Europe and beyond.^1^ This classification is particularly relevant for identifying candidates for biologic treatment in severe, uncontrolled CRSwNP, where type 2 inflammation is already highly prevalent.^1^, ^31^ However, it is important to acknowledge that this dichotomous classification may not fully capture the complex pathophysiology of primary diffuse CRS. The specificity of a single elevated systemic Th2 biomarker—such as blood eosinophils ≥150/μL or total serum IgE >100 kU/L—to identify type 2 inflammation in the sinuses remains controversial. These relatively low cut‐off values, however, improve sensitivity for detecting type 2 inflammation in severe uncontrolled CRSwNP, where the a priori probability of type 2 inflammation is already high. Moreover, in our cohort, patients classified as type 2 CRSwNP more frequently presented with comorbid asthma, allergic rhinitis and prior sinus surgery than non‐type 2 CRSwNP, supporting the presence of underlying Th2‐driven disease. A positive likelihood ratio of 3.5 for comorbid asthma and 3.7 for comorbid allergic rhinitis indicates that patients with CRSwNP and a comorbid type 2 disease are approximately 3.5 times more likely to exhibit type 2 inflammation than those without.

It is also noteworthy that current biomarkers do not reliably predict the therapeutic response to biologics.^32^, ^33^ Therefore, refinement of classification criteria and improved stratification of patients remains important. Our study aims to contribute to this process by examining symptom profiles across current endotypes, offering potential insights into CRS pathophysiology.

However, our study has some methodological limitations. As all participants were referred for surgery due to uncontrolled disease, a selection bias cannot be excluded. Together with the current lower blood eosinophil threshold, this may explain the higher proportion of patients being classified as type 2 in our cohort compared to previous research.^4^, ^6^ We also found that non‐type 2 CRSwNP patients have better HRQoL, so they may require surgery less often, which could potentially lead to their underrepresentation in our study. However, it has been shown that patients electing medical management have comparable sinus‐specific symptoms.^34^ Interobserver variability in NPS assessments cannot be excluded, as endoscopic examinations were not video‐documented and grading was not performed by multiple examiners in a blinded setting. Nonetheless, previous studies have demonstrated substantial interrater agreement among academic rhinologists in nasal polyp scoring, suggesting that reproducibility in our study is likely acceptable.^35^, ^36^

## 5 CONCLUSION

The endotype of CRSwNP significantly affects symptom severity and quality of life, with patients with type 2 CRSwNP experiencing worse sinonasal symptoms and reduced HRQoL. Both endotypes induce nasal obstruction. Decreased sense of smell and rhinorrhea are major symptoms of CRS but are particularly prominent in patients with type 2 CRSwNP. Both of these symptoms might provide endotypical and therefore pathophysiological information. Further studies, including real‐world analyses, are needed to confirm these findings and establish their relevance in defining key outcomes for future interventional trials, particularly those evaluating biologic therapies.

## AUTHOR CONTRIBUTIONS

**Stijn Bogaert**: Conceptualization; formal analysis; writing—original draft; investigation; validation; software; visualization. **Elisabeth Rheindorf**: Data curation; investigation; writing—original draft. **Stefan Dazert**: Investigation; supervision. **Stefan Volkenstein**: Conceptualization; supervision. **Lisa Knipps**: Writing—review and editing; validation. **Jonas Jae‐Hyun Park**: Conceptualization; methodology. **Oliver Pfaar**: Supervision; writing—review and editing.

## CONFLICT OF INTEREST STATEMENT

Dr. Pfaar reports grants and/or personal fees and/or travel support from AEDA, Alfried Krupp Krankenhaus, ALK‐Abelló, Allergopharma, Almirall, Altamira Therapeutics, ASIT Biotech, AstraZeneca, Bencard Allergie GmbH/Allergy Therapeutics, Blueprint, Cliantha, Deutsche AllergieLiga e.V., Deutsche Forschungsgemeinschaft, Dustri‐Verlag, ECM Expro&Conference Management GmbH, Forum für Medizinische Fortbildung, Georg‐Thieme‐Verlag, GSK, HAL Allergy Holding B.V./HAL Allergie GmbH, Inmunotek, Ingress Health, Institut für Disease Management, IQVIA Commercial, Japanese Society of Allergology, Königlich Dänisches Generalkonsulat, Laboratorios LETI/LETI Pharma, Lilly, Lofarma, Medizinische Hochschule Hannover, med update europe GmbH, Meinhardt Congress GmbH, Novartis, Paul‐Ehrlich‐Institut, Paul‐Martini‐Stiftung, PneumoLive, Pohl‐Boskamp, Procter & Gamble, Red Maple Trials Inc., RG Arztefortbildung, ROXALL Medizin, Sanofi Aventis, Sanofi Genzyme, Springer Publisher, Stallergenes Greer, streamedup! GmbH, Technical University Dresden, Wiley Publishers, Wort & Bild Verlag, Verlag ME; outside the submitted work, Oliver Pfaar is Vice President of the European Academy of Allergy and Clinical Immunology (EAACI), a member of EAACI Excom as well as a member of the external board of directors of the German Society of Allergy and Clinical Immunology (DGAKI); coordinator, main author or co‐author of different position papers and guidelines in rhinology, allergology, and allergen‐immunotherapy; and he is Editor‐in‐Chief of Clinical Translational Allergy and Associate Editor of Allergy. Dr. Knipps reports grants and/or personal fees and/or travel support from AstraZeneca, BioCryst, CSL Behring, GlaxoSmithKline, RG Arztefortbildung, Sanofi‐Aventis and Sanofi‐Genzyme, Takeda Pharma. The other authors declare that they have no competing interests.

## CONSENT FOR PUBLICATION

Not applicable.

## Supporting information

Figure S1

Table S1

## Data Availability

The datasets generated and/or analyzed during the current study are available in the Mendeley data repository, https://data.mendeley.com/datasets/3jgw78r6k2/1.
