# Supplementary figures and images for: Quality of life in type 2 and non‐type 2 endotypes in chronic rhinosinusitis with nasal polyps: A prospective trial
Source: Clin Transl Allergy. 2025 May 31;15(6):e70070. doi: 10.1002/clt2.70070 (PMC12126119; doi:10.1002/clt2.70070)

**Supplementary Figure 1.** Correlation between age and facial pain/pressure in type 2 CRSwNP (p = 0.01, ρ = -0,25).

**
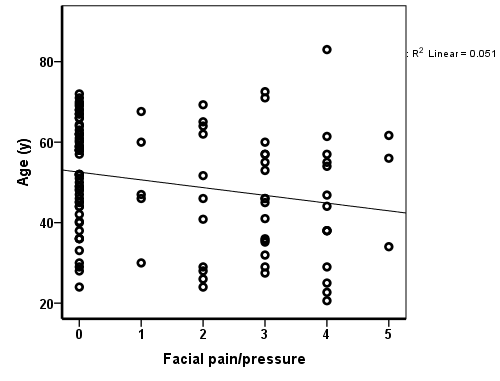
**

Supplement: Supplementary file 1 — Figure S1 [file CLT2-15-e70070-s002.docx]
