# Supplementary material for: Quality of life in type 2 and non‐type 2 endotypes in chronic rhinosinusitis with nasal polyps: A prospective trial
Source: Clin Transl Allergy. 2025 May 31;15(6):e70070. doi: 10.1002/clt2.70070 (PMC12126119; doi:10.1002/clt2.70070)
Supplement: Supplementary file 2 — Table S1 [file CLT2-15-e70070-s001.docx]

**Supplementary Table 1.** Percentage of positive outcomes of the separate CS and PNS symptom scores.

| Symptoms | Type 2 | Non-type 2 | P-value |
| --- | --- | --- | --- |
| Nasal obstruction | 94.7% | 88.9% | 0.4 |
| Rhinorrhea | 63.7% | 0.0%­ | <0.001 |
| Postnasal drip | 64.6% | 55.6% | 0.7 |
| Reduced sense of smell | 83.2% | 44.4% | 0.01 |
| Facial pain / pressure | 43.4% | 11.1% | 0.08 |
| Sneezing | 69.9% | 55.6% | 0.5 |
| Thick nasal discharge | 79.5% | 77.8% | 0.9 |

A sympom score of ≥2 was considered as a positive outcome. *CS* cardinal symptoms of CRS, *PNS* primary nasal symptoms.
